# Statistical declarations versus scientific inferences and clinical judgments: the association of Glucagon-like peptide-1 receptor agonist use with the risk of biliary disease

**DOI:** 10.3389/fendo.2024.1367158

**Published:** 2024-01-29

**Authors:** David E. Most

**Affiliations:** School of Education, Colorado State University, Fort Collins, CO, United States

**Keywords:** biliary disease, GLP-1, gastrointestinal adverse events, scientific inference and reasoning, statistical inference abuse, meta-analytic thinking

## Introduction

The purpose of this piece is to offer a constructive critique of the interpretation of some research findings recently shared by Sohdi et al. in the Journal of the American Medical Association regarding the associations between gastrointestinal adverse events and the use of Glucagon-like peptide 1 (GLP-1) agonists for weight loss in a clinical setting (1). In their interesting and important study, Sodhi et al. obtained data from a random sample from a very large health claims database in order to explore and model the associations between the use of GLP-1 agonists (compared to bupropion-naltrexone) and gastrointestinal adverse events (biliary disease, pancreatitis, bowel obstruction, and gastroparesis). The key quantities of interest are hazard ratios (HR) that characterize the relationship of GLP-1 agonists with each gastrointestinal adverse event relative to the use of bupropion-naltrexone. The claim is made, both in the Results and Discussion, that the use of GLP-1 agonists was not associated with biliary disease. The problem with this claim is that the evidence does not seem to support such a conclusion.

## Evidence and interpretation

What does the evidence seem to indicate? HR point and interval estimates of the relationship between the use of GLP-1 agonists and each of the four gastrointestinal adverse events are presented in the Results and an accompanying Table. The HR point estimate for the relationship between biliary GLP-1 agonist use and biliary disease is 1.50, which indicates that use *was* associated with a 50% higher risk of biliary disease. However, the prose offered in the Results and Discussion explicitly indicates that the use of GLP-1 agonists was not associated with increased risk (1).

Why is there a discrepancy between the evidence and the prose characterization of the results? The discrepancy is a consequence of a common error in interpretation. The mistake is to conflate a binary statistical declaration with a scientific/clinical conclusion. In particular, a declaration of no statistically significant association is conflated with a clinical conclusion that no evidence was found for an association or simply of “no association”. The interpretation of the results, as presented, is based entirely on a binary declaration regarding significance, or equivalently, whether or not a 95% CI for a HR includes 1.00, rather than on the scientific meaning and clinical importance of the magnitude of the estimated association. It is inappropriate to conclude that there is no association because of a binary statistical decision (2). And, therefore, it is inappropriate to conclude that “use of GLP-1 agonists for weight loss compared with use of bupropion-naltrexone was associated with increased risk of pancreatitis, gastroparesis, and bowel obstruction but not biliary disease” (1).

What about uncertainty in the estimates of the associations? The presentation of a 95% confidence interval (CI) for each association is appreciated and helpful for quantifying uncertainty in estimates of relative risk. For all gastrointestinal adverse events, the plausible true values of differential risk associated with the use of GLP-1 agonists, relative to the use of bupropion-naltrexone, ranges from something close nil to many times higher. For example, in the case of bowel obstruction, the lower end of the CI is 1.02, which indicates a 2% higher risk, while the upper end of the interval is 17.40, which indicates a risk that is over 1600% higher. Likewise, the range for biliary disease is .89 to 2.53, which indicates a differential risk from 11% lower to over 150% higher. If uncertainty is taken into account, it is arguable that the difference in the ranges of plausible true HR values that are compatible with the data for all four of the gastrointestinal adverse events are clinically indistinguishable. The true risk differential may be negligible or much higher. However, the only interpretation offered is essentially that a confidence interval does or does not span zero difference in risk (an HR of 1.00). It’s a mistake, however, to argue that the difference between “significant” and “not significant” is clinically significant (3). Rather, embracing uncertainty via the CIs means examining the range of plausible true HR values that are compatible with the data, which, in this case, include many values that indicate the use of GLP-1 agonists might be associated with biliary disease with a magnitude similar to many plausible values for other adverse gastrointestinal events. In addition, the magnitude of the association with biliary disease found by Sohdi et al. is similar to the magnitudes found in a recent large systematic review of 76 randomized clinical trials that examined the same association, and the authors of the systematic review unambiguously concluded that the use of GLP-1 agonists *was* associated with higher risk of biliary disease (4).

Another inconsistent interpretation is offered in passing. Without mentioning the magnitude of HR point and interval estimates, Sodhi et al. note in the Results section that exclusion of hyperlipidemia from the analysis did not change the results (1). However, the associated table shows that the 95% CIs for *both* bowel obstruction (.87, 15.10) and biliary disease (.84, 2.51) include 1.00. Using their criteria for making a binary statistical declaration means that the results are *not* the same as when not excluding hyperlipidemia. A more statistically consistent (though substantively incorrect) interpretation would be that, when excluding hyperlipidemia, use of GLP-1 agonists was not associated with increased risk for bowel obstruction. However, the more important observation of clinical sameness is warranted, though the distinction between clinical importance and statistical significance is not made.

## Discussion

The concern described here might be considered an example of a more general century-old problem of not distinguishing between statistical inference and scientific inference (5). Empirical examinations of the literature in various disciplines suggest that associated interpretational errors happen more often than not (2). The interpretation that the use of GLP-1 agonists was not associated with increased risk of biliary disease depends on mistakenly conflating the notion of a declaration regarding statistical significance with a clinical judgement regarding the nature of an association. Instead of focusing on whether or not the true differential risk could be zero, a better way to make meaning of these data might be to offer a substantive interpretation of the magnitude of the relative risk, which brings clinical expertise to bear and that fully embraces statistical and scientific uncertainty. In many clinical settings, a 50% higher risk (and possibly higher) of an adverse outcome would not be considered inconsequential. Both generating cumulative knowledge and optimizing clinical outcomes depend on summaries of findings that have fidelity to the evidence.

## Author contributions

DM: Writing – original draft, Writing – review & editing.
